# Effects of Rumen-Protected Glucose in Diets and Rumen-Protected Taurine Supplementation on the Colonic Mucosal Barrier in Yaks

**DOI:** 10.3390/biology15171526

**Published:** 2026-09-03

**Authors:** Shoupei Zhao, Huaming Yang, Jia Zhou, Mingyu Cao, Bai Xue

**Affiliations:** 1 Animal Nutrition Institute, Sichuan Agricultural University, Chengdu 611130, China; 2023114011@stu.sicau.edu.cn (S.Z.); 13980277829@163.com (H.Y.); jjzhouz@163.com (J.Z.); mycao2021114018@163.com (M.C.); 2 Longkong Town Agricultural Comprehensive Service Center, Leshan 614407, China; 3 Chongqing Academy of Animal Sciences, Chongqing 402460, China

**Keywords:** yak, rumen-protected glucose, rumen-protected taurine, intestinal barrier, colonic microbiota

## Abstract

The intestinal barrier plays a critical role in maintaining gastrointestinal health and nutrient utilization in yaks. This study investigated the effects of dietary RPG level and RPT supplementation on colonic barrier function and microbial composition in yaks. Twenty-eight healthy male yaks (3 years old; 192.7 ± 4.52 kg) were assigned to a 2 × 2 factorial design with two dietary rumen-protected glucose (RPG) levels (1.0% or 3.0% of dietary DM) and two rumen-protected taurine (RPT) supplementation levels (5 or 20 g/animal/day) for 63 days. High-level RPG impaired colonic physical barrier function by reducing tight junction protein expression and microbial diversity, whereas high-level RPT mainly compromised chemical and immune barrier function by decreasing diamine oxidase activity, mucin-2, and secretory immunoglobulin A, accompanied by alterations in the colonic microbial community. Significant interactions between RPG and RPT were observed for several barrier- and microbiota-related indices. Overall, the effects of RPG and RPT on colonic health were dose-dependent, and moderate supplementation, particularly the combination of 1.0% dietary RPG and 5 g/day RPT, was the most effective in maintaining colonic barrier integrity and microbial homeostasis in yaks.

## Simple Summary

Yaks are well adapted to high-altitude environments, but their growth and health are often limited by poor-quality forage and seasonal nutrient shortages. To improve production efficiency, yaks are increasingly raised under intensive feeding systems outside their traditional grazing areas. Changes in environment and management practices may increase the risk of digestive disorders, making hindgut health a key factor in maintaining yak health and productivity. In this study, we investigated whether different dietary levels of protected glucose and protected taurine could improve hindgut health in yaks. We found that high levels of protected glucose negatively affected hindgut health by weakening the physical protection of the intestinal wall and disturbing the balance of beneficial microorganisms. Meanwhile, high levels of protected taurine mainly reduced the ability of the hindgut to maintain its chemical and immune defenses and altered the microbial community. Overall, the effects of these nutrients depended on their dietary levels, and the combination of lower levels of both nutrients showed the most beneficial effects by maintaining hindgut barrier function and microbial balance. These findings provide practical guidance for developing appropriate feeding strategies to improve yak health, production efficiency, and sustainable farming.

## 1. Introduction

Yaks (*Bos grunniens*) are indigenous to the high-altitude regions of the Qinghai–Tibet Plateau and neighboring alpine areas, where they are exposed to chronic hypoxia, low temperatures, and seasonal forage shortages [1]. The global domestic yak population is estimated at approximately 16 million, with more than 95% distributed in China [2]. Yaks are well adapted to these harsh environments and play an important role in pastoral production systems by providing meat, milk, hides, and other products while supporting the livelihoods of local communities [3]. However, traditional yak production relies predominantly on natural grazing, and seasonal declines in forage availability and quality can limit nutrient intake and growth performance [4]. Consequently, concentrate supplementation and intensive fattening systems have increasingly been adopted to improve production efficiency [5]. The associated changes in diet, housing, transportation, and management may disrupt gastrointestinal homeostasis and increase the risk of intestinal dysfunction [6,7]. Because the intestine is a major site of nutrient absorption and an important interface among the host, diet, and gut microbiota, maintaining intestinal barrier integrity, immune homeostasis, and microbial balance is essential for efficient nutrient utilization and animal productivity.

Rumen-protected technology can reduce the degradation of functional nutrients by ruminal microorganisms and increase their availability to post-ruminal tissues. Glucose is an important metabolic substrate for intestinal epithelial and immune cells. However, dietary glucose is extensively utilized by ruminal microorganisms, which may limit its direct availability to post-ruminal tissues. Rumen-protected glucose (RPG) can increase post-ruminal glucose availability, and previous studies have shown that RPG supplementation may improve energy status and influence intestinal immune function and gut microbial communities [8,9,10]. Taurine, in contrast, is involved in bile acid conjugation, antioxidant defense, and immune regulation. Rumen-protected taurine (RPT) may increase the availability of taurine beyond the rumen, while taurine supplementation has been reported to promote intestinal development, alleviate intestinal injury, reduce oxidative stress and inflammation, enhance immune function, and regulate gut microbial composition and function [11,12,13]. Thus, RPG and RPT may have complementary nutritional effects, with RPG primarily supporting metabolic energy supply and RPT contributing to antioxidant, anti-inflammatory, immunomodulatory, and intestinal-protective functions.

Importantly, biological responses to these functional nutrients may vary with dietary level. Our previous studies showed that a high dietary glucose level (3% of dietary dry matter (DM)) improved growth performance compared with a low level (1% of dietary DM) in yaks [14]. Similarly, a high-level taurine supplementation level (15 g/d) reduced body-weight loss during transportation compared with a low level (5 g/d) [15]. However, higher supplementation levels do not necessarily produce proportionally greater benefits. For example, high-glucose diets have been reported to alter the gut microbiota and increase intestinal permeability in mice [16]. Similarly, excessive taurine supplementation adversely affected growth performance and hepatic and intestinal health in piglets [17]. These findings indicate that the effects of functional nutrients may be dose-dependent and that excessive supplementation may compromise intestinal or metabolic homeostasis. Nevertheless, the intestinal responses of yaks to different dietary levels of RPG and RPT remain poorly understood, particularly with respect to intestinal barrier integrity, antioxidant and immune status, inflammatory responses, and gut microbial homeostasis.

Therefore, we hypothesized that appropriate dietary levels of RPG and RPT would maintain or improve intestinal homeostasis, whereas higher levels might attenuate these beneficial effects or induce unfavorable gastrointestinal responses. To test this hypothesis, a 2 × 2 factorial experiment was conducted in yaks under intensive feeding conditions, with RPG supplied at 1% or 3% of dietary DM and RPT at 5 or 20 g/animal/day. The effects of these dietary treatments on growth performance and yak intestinal health were evaluated by systematically assessing multiple components of the intestinal barrier, including the physical barrier (tight junction protein expression and diamine oxidase activity and content), chemical barrier (mucin-2 and lysozyme), immune barrier (secretory immunoglobulin A, inflammatory cytokines, and inflammation-related signaling pathways), and gut microbial community. These findings may help identify appropriate supplementation levels of RPG and RPT and provide a scientific basis for optimizing dietary formulations and nutritional management in translocated intensive yak-fattening systems.

## 2. Materials and Methods

### 2.1. Ethics Statement

All animal procedures were conducted in accordance with the Chinese Guidelines for Animal Welfare and were approved by the Experimental Animal Ethics Committee of the Animal Nutrition Institute, Sichuan Agricultural University (approval no. 20210628, approval date 12 November 2021).

### 2.2. Animals, Experimental Design, and Management

The experiment was conducted from July to September 2023 at the Research and Teaching Farm of Sichuan Agricultural University, Ya’an, China (30.0° N, 103.0° E). Twenty-eight healthy 3-year-old male Maiwa yaks with similar initial body weights (192.7 ± 4.52) were selected and randomly assigned to four treatment groups (*n* = 7), with one animal serving as an experimental unit. All yaks were individually tethered in separate pens throughout the experimental period. Based on our previous findings [14,15], a 2 × 2 factorial design was used to evaluate the effects of dietary RPG and RPT. RPG was incorporated into the total mixed ration at 1.0% (low RPG, LG) or 3.0% (high RPG, HG) of dietary dry matter (DM), with the proportions of other dietary ingredients adjusted accordingly to maintain similar nutrient composition among diets. RPT was provided as an additional feed additive at 5 g/animal/day (low RPT, LT) or 20 g/animal/day (high RPT, HT), without replacing any basal diet ingredient. Thus, the four treatments were 1.0% RPG + 5 g/day RPT (LGLT), 1.0% RPG + 20 g/day RPT (LGHT); 3.0% RPG + 5 g/day RPT (HGLT), and 3.0% RPG + 20 g/day RPT (HGHT). The diet was formulated according to NY/T 815–2004 [18], and their ingredient composition and nutrient levels are presented in Table 1. The RPG product (Meiweineng; Shanghai MNC Bio-Tech Co., Ltd., Shanghai, China) contained 50% glucose, with an 80% ruminal bypass rate and a 90% intestinal release rate, whereas the RPT product (Qingdao Kaillide Biotechnology Co., Ltd., Qingdao, China) contained 50% taurine, with the same ruminal bypass and intestinal release rates. Both products were provided in solid form. All yaks were individually tethered in separate pens throughout the 77-day experiment, which included a 14-day adaptation period and a 63-day experimental period. Before the trial, all animals were dewormed, individually ear-tagged, and housed in disinfected pens. Yaks were fed twice daily at 09:00 and 18:00 and had free access to fresh drinking water. During feed preparation, RPG was incorporated into the concentrate at the designated levels, whereas RPT was thoroughly mixed with approximately 15% of the ration allocated for each meal and offered first to ensure complete consumption before the remaining ration was provided. Each yak was weighed after fasting on days 0 and 90, and dry matter intake (DMI) was recorded daily. The feed-to-gain ratio (F/G) was calculated accordingly.

### 2.3. Analysis of Dietary Nutrient Composition

The nutrient composition of feed samples was determined following the methods of Zhao et al. [5]. Briefly, feed and fecal samples were dried at 65 °C for 48 h, weighed, and ground through a 1 mm sieve. DM and ash were determined according to AOAC (2005; methods 930.15 [20] and 942.05 [20]). N was analyzed by the Kjeldahl method (2005, AOAC 981.10 [20]) and expressed as crude protein (CP; N × 6.25). Ether extract (EE) was determined by Soxhlet extraction (1990, AOAC 920.39 [21]). Calcium (Ca) and phosphorus (P) were determined by atomic absorption spectrometry and fluorescence spectrophotometry, respectively (1990, AOAC 985.35 [21] and 986.24 [21]). Neutral detergent fiber (NDF) and acid detergent fiber (ADF) were analyzed according to the method of Van Soest et al. [22]. Acid-insoluble ash (AIA) was determined according to the method of Van Keulen et al. [23].

### 2.4. Collection and Analysis of Colonic Mucosa

At the end of the experiment, all yaks were humanely slaughtered for sample collection. Immediately after slaughter, each intestinal segment was ligated to prevent luminal contamination, and the attached mesenteric fat was carefully removed. A middle section of the colon was excised and gently rinsed with sterile physiological saline to remove intestinal contents. The colonic mucosa was then gently and uniformly scraped using sterile glass microscope slides. The collected mucosal samples were transferred into sterile 2 mL cryogenic tubes, immediately snap-frozen in liquid nitrogen, and subsequently stored at −80 °C until further analysis.

The concentrations of secretory immunoglobulin A (sIgA), lysozyme (LZM), and mucin-2 (MUC2), together with the activity of diamine oxidase (DAO), in the colonic mucosa were determined using an enzyme-linked immunosorbent assay (ELISA). Prior to analysis, mucosal samples were thoroughly ground in liquid nitrogen and homogenized. The resulting homogenates were analyzed using bovine-specific ELISA kits (Jiangsu Meimian Industrial Co., Ltd., Yancheng, China) according to the manufacturer’s instructions.

For RNA extraction and quantitative real-time PCR analysis, total RNA was extracted from colonic mucosal samples using TRIzol reagent (Invitrogen, Carlsbad, CA, USA) according to the manufacturer’s instructions. Briefly, approximately 30–50 mg of colonic mucosa was ground in liquid nitrogen, homogenized in 1 mL of TRIzol reagent, and subjected to phase separation with chloroform. RNA was precipitated with isopropanol, washed three times with pre-cooled 75% ethanol, air-dried, and dissolved in RNase-free water. The purity and concentration of the extracted RNA were determined using a NanoDrop 2000 spectrophotometer (Thermo Fisher Scientific, Wilmington, DE, USA). RNA samples with an OD260/OD280 ratio between 1.8 and 2.1 were considered acceptable for subsequent analysis. First-strand complementary DNA (cDNA) was synthesized from total RNA using the ExonScript RT SuperMix with dsDNase kit (ExonGene, Shanghai, China) according to the manufacturer’s instructions. The resulting cDNA was stored at −80 °C until further analysis. The mRNA expression levels were quantified by quantitative real-time PCR (qPCR). Gene-specific primers were designed based on mRNA sequences obtained from the NCBI GenBank database and synthesized by Sangon Biotech Co., Ltd. (Chengdu, China). The primer sequences are listed in Table 2. Quantitative PCR was performed in a 10 μL reaction volume containing 5 μL of 2× UltraStart SYBR Green qPCR Master Mix, 0.2 μL each of the forward and reverse primers, 0.2 μL of 50× ROX Reference Dye II, 1 μL of the cDNA template, and PCR-grade water to the final volume. Amplification was carried out under the following cycling conditions: initial denaturation at 95 °C for 3 min, followed by 40 cycles of denaturation at 95 °C for 5 s and annealing/extension at 60 °C for 30 s, with a final dissociation stage consisting of 95 °C for 15 s and 60 °C for 60 s. All samples were analyzed in quadruplicate. Relative gene expression levels were calculated using the 2^−ΔΔCt^ method, with *β-actin* serving as the internal reference gene.

### 2.5. Colonic Digesta Collection and 16S rRNA Gene Sequencing

Approximately 30 g of colonic digesta was aseptically collected from the middle section of the colon immediately after slaughter, aliquoted into four sterile 5 mL RNase-/DNase-free cryogenic tubes, snap-frozen in liquid nitrogen, and stored at −80 °C until microbial analysis. Microbial genomic DNA was extracted using a DNA Extraction Kit (Tiangen Biotech Co., Ltd., Beijing, China) according to the manufacturer’s instructions and further purified using the ZymoBIOMICS DNA Microprep Kit (Cat. No. D4301, Zymo Research, Irvine, CA, USA). DNA integrity was assessed by 0.8% agarose gel electrophoresis, and DNA concentration was quantified using the PicoGreen assay on a Tecan Infinite F200 microplate reader (Tecan Group Ltd., Männedorf, Switzerland).

The V4 hypervariable region of the bacterial 16S rRNA gene was amplified using the universal primers 515F (5′-GTGYCAGCMGCCGCGGTAA-3′) and 806R (5′-GGACTACHVGGGTWTCTAAT-3′), with sample-specific index sequences. PCR amplification was performed under standard conditions, with each sample amplified in triplicate. PCR products from the same sample were pooled, verified by 2% agarose gel electrophoresis, purified using the Zymoclean Gel Recovery Kit (Cat. No. D4008, Zymo Research), and quantified using a Qubit 2.0 Fluorometer (Thermo Fisher Scientific, Waltham, MA, USA). Purified amplicons were pooled in equimolar concentrations. Sequencing libraries were constructed using the MGIEasy Universal DNA Library Prep Kit (Cat. No. MGI#1000005282; MGI Tech Co., Ltd., Shenzhen, China), and paired-end sequencing (PE250) was performed using the DNBSEQ-G99RS High-Throughput Sequencing Reagent Kit (MGI Tech Co., Ltd., Shenzhen, China).

### 2.6. Bioinformatics and Statistical Analysis

Raw paired-end sequencing reads were first merged using FLASH (v1.2.11). Sample-specific sequences were subsequently demultiplexed according to their barcode sequences using SABRE (https://github.com/najoshi/sabre, 17 May 2024), and the barcode sequences were removed. Quality filtering was performed using QIIME 2 (v2020.2). Sequences with an average quality score <30, a length <200 bp, or more than 0 ambiguous bases (N) were removed. The resulting high-quality sequences were denoised and screened for chimeric sequences using the DADA2 (https://benjjneb.github.io/dada2/, 17 May 2024) algorithm implemented in QIIME 2, generating an amplicon sequence variant (ASV) feature table and representative feature sequences. Taxonomic classification of the ASV sequences was performed using a Naïve Bayes classifier trained on the SILVA 132 database. Multiple sequence alignment of the representative ASV sequences was conducted using QIIME 2, and a phylogenetic tree was constructed using the FastTree plugin (https://morgannprice.github.io/fasttree/, 17 May 2024). To ensure comparability among samples, the ASV feature table was rarefied to an equal sequencing depth based on the sample with the lowest number of sequences. Alpha diversity indices were calculated using R software (v4.0.5). The phylogenetic diversity (PD) index was calculated using the Picante package (R software, v4.0.5), whereas other diversity indices were determined using the Vegan package (R software, v4.0.5). Beta diversity analyses were conducted based on different distance matrices. Unweighted and weighted UniFrac distances were calculated using the GUniFrac package (R software, v4.0.5), while Bray–Curtis and Jaccard distances were calculated using the vegan package. Principal coordinate analysis (PCoA) was performed using the ape package (R software, v4.0.5).

Statistical analyses were performed using SPSS 20.0 (IBM Corp., Armonk, NY, USA). A two-way analysis of variance was conducted using the General Linear Model (GLM) procedure in SPSS according to a 2 × 2 factorial design to evaluate the main effects of dietary RPG level and RPT supplementation level, as well as their interaction (RPG × RPT). When significant interaction effects were detected, differences among treatment groups were further evaluated using one-way ANOVA. Multiple comparisons were performed using Duncan’s multiple range test. All data are presented as means ± standard error of the mean (SEM). Statistical significance was declared at *p* < 0.05, and highly significant differences were considered at *p* < 0.01.

## 3. Results

### 3.1. Effects of RPG and RPT on Growth Performance

DMI, ADG, and F/G were not significantly affected by dietary RPG level or RPT supplementation level, and no significant RPG × RPT interaction was observed (*p* > 0.05) (Table 3).

### 3.2. Effects of RPG and RPT on Colonic Physical Barrier Function

A significant interaction between RPG and RPT was observed for the mRNA expression of *occludin* in the colonic mucosa of yaks (*p* < 0.05) (Table 4). Regarding the main effect of dietary RPG, a high dietary RPG level significantly decreased the relative mRNA expression of *JAM-A*, *Claudin-1*, and *Claudin-4* (*p* < 0.01), and tended to reduce *ZO-1* expression (*p* = 0.087). Regarding the main effect of RPT supplementation, a high level of RPT significantly decreased the mRNA expression of *Claudin-1* (*p* < 0.01) and tended to reduce *JAM-A* expression (*p* = 0.051).

**Figure 1:**
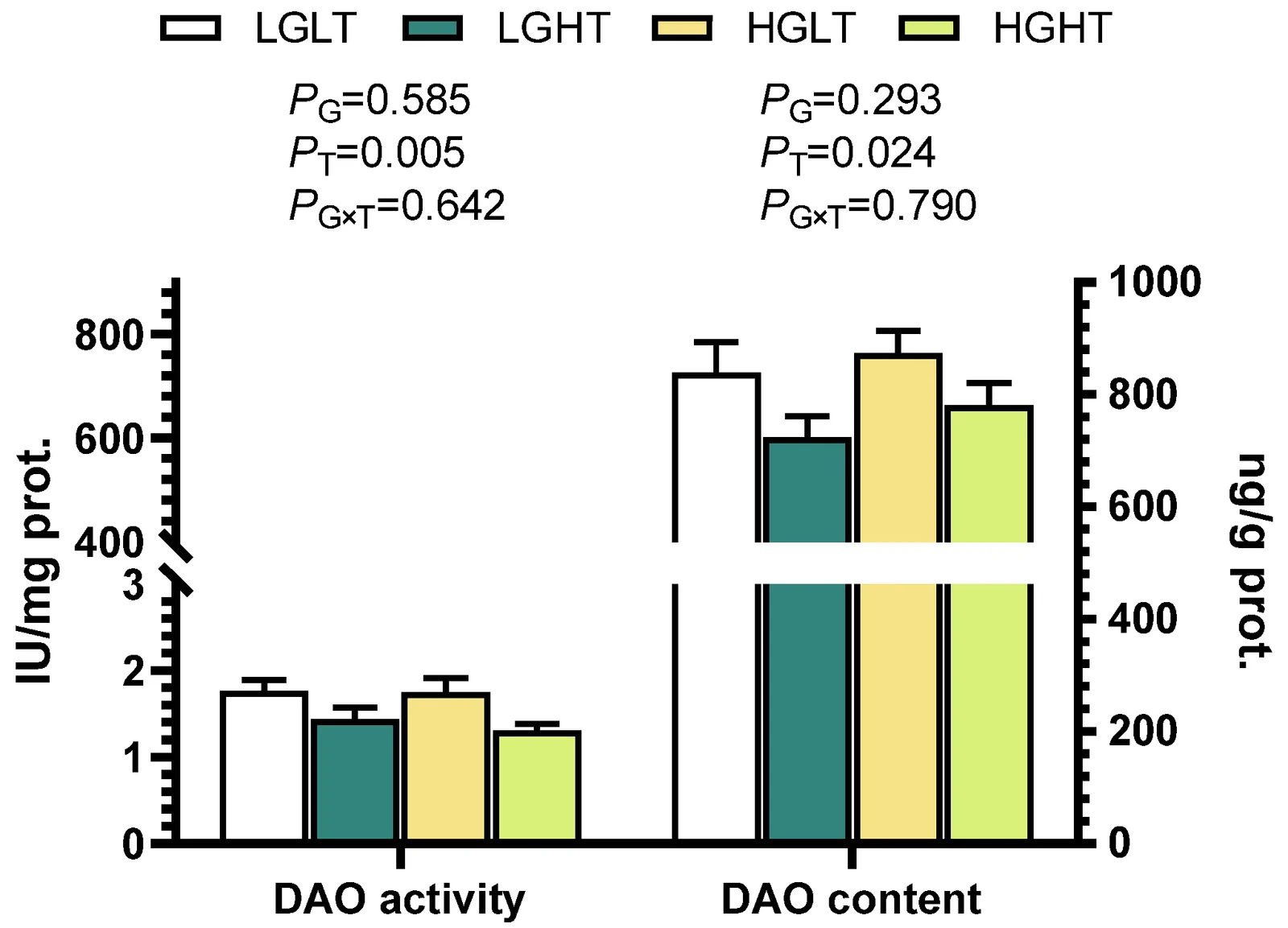
Effects of RPG and RPT on the DAO activity and content of colonic in yaks. prot. = protein; LGLT = 1.0% RPG + 5 g/day RPT; LGHT = 1.0% RPG + 20 g/day RPT; HGLT = 3.0% RPG + 5 g/day RPT; HGHT LT = 3.0% RPG + 20 g/day RPT. *P*_G_, main effect of dietary RPG level; *P*_T_, main effect of RPT supplementation level; *P*_G×T_, interaction between dietary RPG level and RPT supplementation level.

Dietary RPG level had no significant effect on DAO activity or content in the colonic mucosa (*p* > 0.05) (Figure 1). In contrast, increasing the level of RPT supplementation significantly decreased DAO activity (*p* < 0.01) and content (*p* < 0.05).

### 3.3. Effects of RPG and RPT on Colonic Chemical Barrier Function

**Figure 2:**
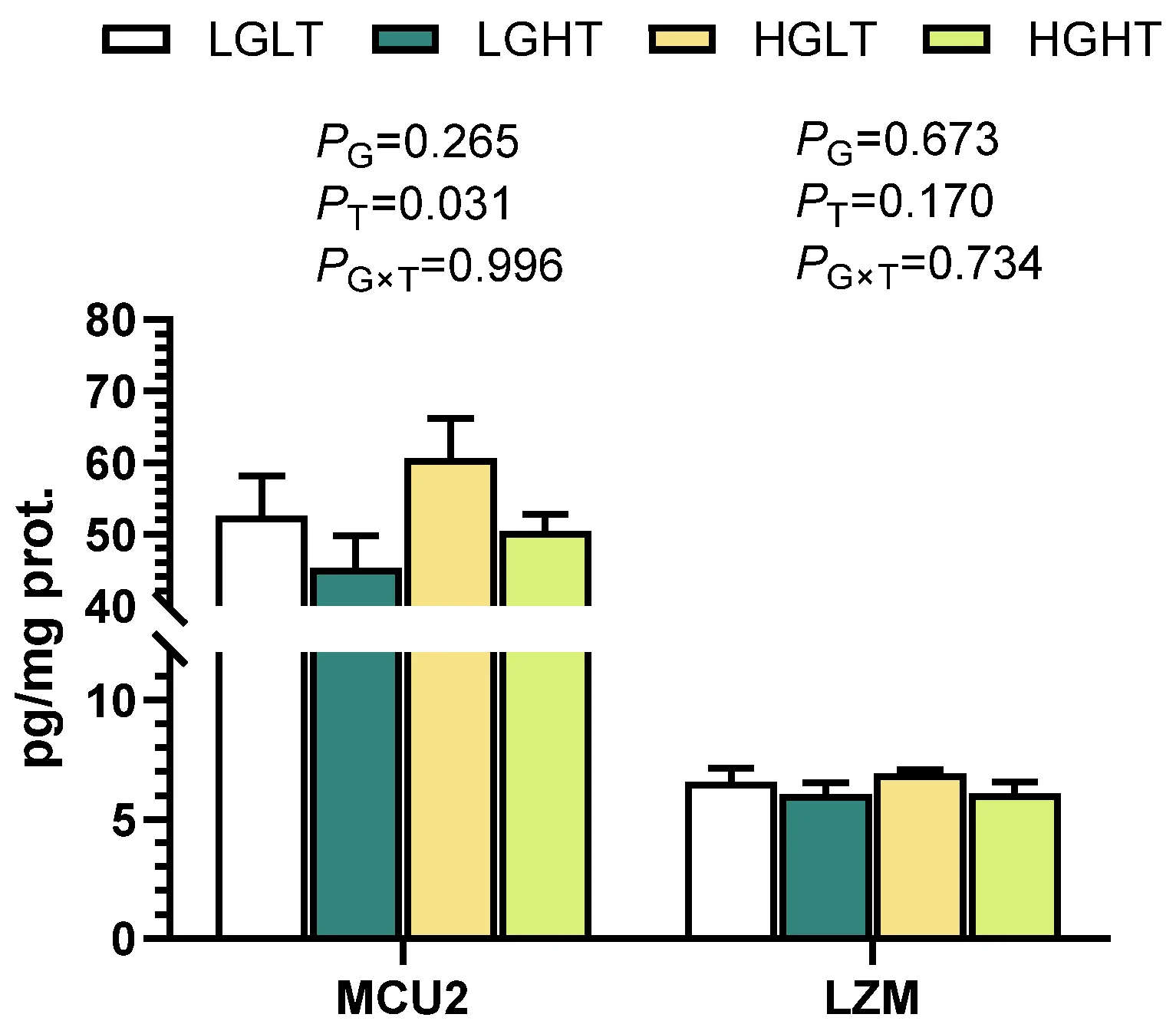
Effects of RPG and RPT on the MCU2 and LZM content of colonic in yaks. prot. = protein; LGLT = 1.0% RPG + 5 g/day RPT; LGHT = 1.0% RPG + 20 g/day RPT; HGLT = 3.0% RPG + 5 g/day RPT; HGHT LT = 3.0% RPG + 20 g/day RPT; MUC2 = mucin-2; LZM = lysozyme. *P*_G_, main effect of dietary RPG level; *P*_T_, main effect of RPT supplementation level; *P*_G×T_, interaction between dietary RPG level and RPT supplementation level.

Increasing dietary RPT levels significantly decreased the mucosal content of MUC2 in the colon (*p* < 0.05) (Figure 2). No significant interaction effect between RPG and RPT was observed on the concentrations of MUC2 and LZM in the colonic mucosa.

### 3.4. Effects of RPG and RPT on Colonic Immune Barrier Function

**Figure 3:**
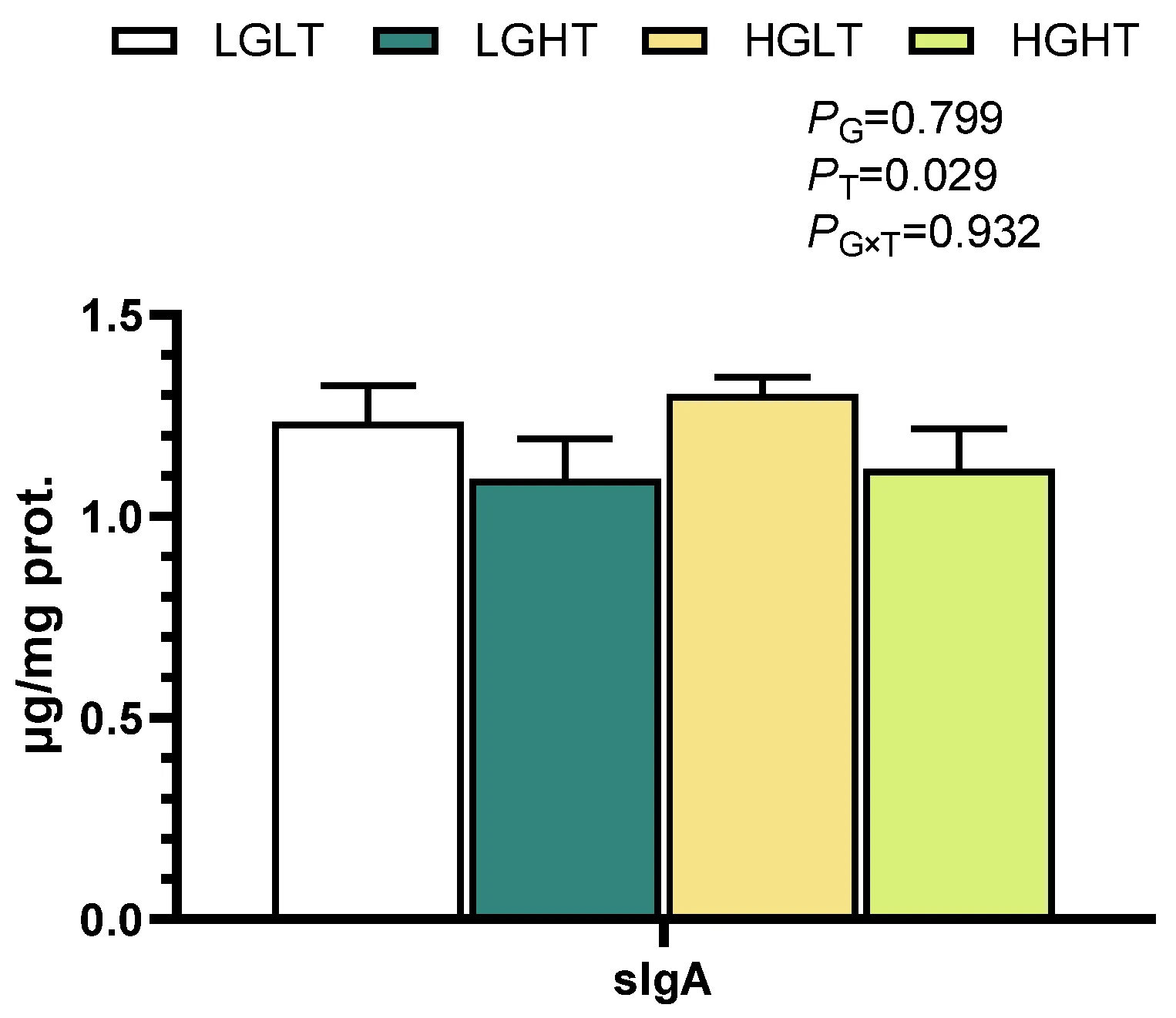
Effects of RPG and RPT on the sIgA content of colonic in yaks. prot. = protein; LGLT = 1.0% RPG + 5 g/day RPT; LGHT = 1.0% RPG + 20 g/day RPT; HGLT = 3.0% RPG + 5 g/day RPT; HGHT LT = 3.0% RPG + 20 g/day RPT; sIgA = secretory immunoglobulin A. *P*_G_, main effect of dietary RPG level; *P*_T_, main effect of RPT supplementation level; *P*_G×T_, interaction between dietary RPG level and RPT supplementation level.

Increasing dietary RPG levels significantly decreased the content of sIgA in the colonic mucosa of yaks (*p* < 0.05) (Figure 3). Similarly, high-level RPT supplementation significantly reduced sIgA in the colonic mucosa (*p* < 0.05). No significant interaction between RPG and RPT was observed for sIgA concentration in the colonic mucosa (*p* > 0.05).

High dietary RPG levels significantly decreased the mRNA expression of *JNK* and *NF-κB* in the colonic mucosa (*p* < 0.01) (Table 4). Similarly, high-level RPT supplementation significantly decreased the mRNA expression of *JNK* and *NF-κB* in the colonic mucosa (*p* < 0.01). A significant interaction between RPG and RPT was detected for *IL-10* mRNA expression (*p* < 0.01). Higher dietary RPG levels reduced the mRNA expression of *TNF-α*, *IL-6*, and *IL-1β* (*p* < 0.05), whereas high-level RPT supplementation decreased the mRNA expression of *IL-6* and *IL-1β* (*p* < 0.05).

### 3.5. Effects of RPG and RPT on Colonic Microbial Barrier

**Figure 4:**
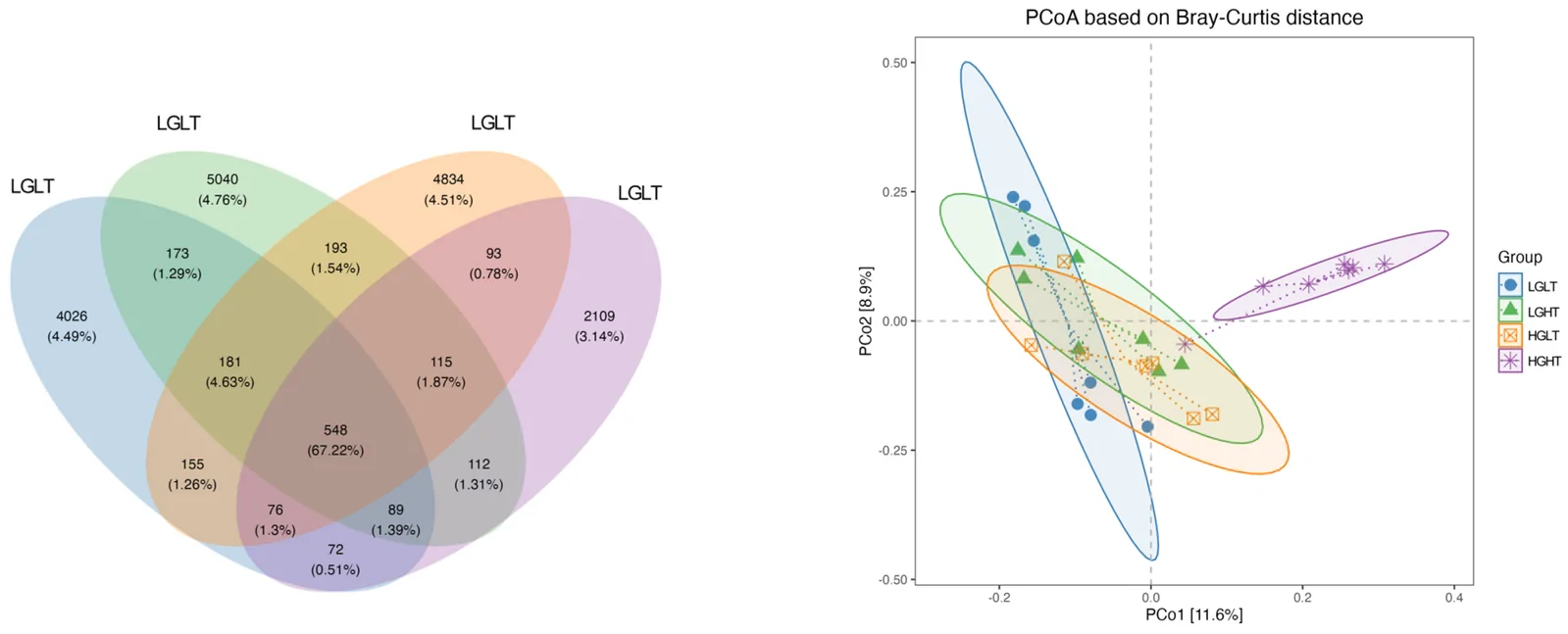
Effects of RPG and RPT on shared and unique ASVs and β-diversity of the colonic microbiota in yaks. LGLT = 1.0% RPG + 5 g/day RPT; LGHT = 1.0% RPG + 20 g/day RPT; HGLT = 3.0% RPG + 5 g/day RPT; HGHT LT = 3.0% RPG + 20 g/day RPT; ASVs = amplicon sequence variants.

The ASV abundance is shown in Venn diagram in Figure 4. The ASVs shared by all groups was 548, and the total ASVs in the LGLT, LGHT, HGLT, and HGHT groups were 4026, 5040, 4834, and 2109, respectively (Figure 4).

A high dietary RPG level significantly decreased the alpha diversity indices of the colonic microbiota, including observed species, Chao1, ACE, and Simpson (*p* < 0.05), and tended to decrease the PD index (*p* = 0.055) (Table 5). High-level RPT supplementation tended to decrease the observed species, Chao1, and ACE indices (*p* = 0.087, 0.076, and 0.074, respectively). No significant interactions between RPG and RPT were observed for any alpha diversity index (*p* > 0.05).

At the phylum level, Firmicutes exhibited the highest relative abundance in the colonic microbiota, followed by Bacteroidetes, and these two phyla together accounted for approximately 73% of the total microbial abundance, whereas the relative abundances of other phyla were below 1%. A high dietary RPG level significantly decreased the relative abundance of Proteobacteria (*p* < 0.05), while high-level RPT supplementation significantly increased the relative abundance of Verrucomicrobia (*p* < 0.05) (Table 6). At the genus level, the relative abundances of *Ruminococcaceae UCG-005* were significantly affected by dietary RPG level, with higher abundances observed in the high-RPG group than in the low-RPG group (*p* < 0.05). In addition, a high dietary RPG level significantly decreased the relative abundance of the *Christensenellaceae R-7 group* (*p* < 0.05). Regarding the effect of RPT supplementation, the relative abundances of the *Christensenellaceae R-7 group* were significantly higher in the high-RPT group than in the low-RPT group (*p* < 0.01). Significant interactions between RPG and RPT were observed for the relative abundances of *Ruminococcaceae UCG-010*, *Rikenellaceae RC9 gut group*, and *Bacteroides* in the colonic microbiota (*p* < 0.05).

## 4. Discussion

### 4.1. Effects of RPG and RPT on Growth Performance in Yaks

In the present study, neither RPG nor RPT significantly affected yak growth performance, as reflected by ADG, DMI, and F/G. This finding differs from our previous studies, in which a high dietary glucose level (3% of dietary DM) promoted growth performance compared with a low level (1% of dietary DM) in yaks [14], while a high taurine supplementation level (15 g/d) reduced body-weight loss during transportation compared with a low level (5 g/d) [15]. Together, these findings suggest that the biological effects of glucose and taurine may depend on supplementation level and physiological context. Importantly, previous studies have suggested that high levels of glucose or taurine supplementation may adversely affect intestinal health, and in some cases, compromise growth performance [16,17]. However, previous studies have primarily focused on short-term supplementation or responses under specific stress conditions, whereas the consequences of prolonged supplementation under sustained feeding conditions remain poorly understood. Therefore, the present study was designed to evaluate the long-term effects of RPG and RPT supplementation in yaks. In particular, the physical, chemical, immune, and microbial barriers of the colon were systematically characterized to determine whether prolonged supplementation alters hindgut health and to provide a more comprehensive understanding of the intestinal consequences of long-term RPG and RPT exposure.

### 4.2. Effects of RPG and RPT on Colonic Physical Barrier Function in Yaks

The intestinal physical barrier is composed primarily of the mucus layer, epithelial cells, and intercellular junctions [24]. Tight junctions (TJs), which regulate paracellular transport, are essential for maintaining intestinal barrier integrity [25]. Disruption of TJ proteins increases epithelial permeability and compromises intestinal homeostasis [26]. Glucose availability is important for TJ integrity, and glucose deprivation has been shown to impair epithelial barrier function in yak ruminal epithelial cells [27]. Consistently, supplementation with 200 g/day RPG has been reported to increase occludin expression in the jejunal mucosa of dairy cows [28]. In contrast, high-level RPG supplementation in the present study significantly decreased the expression of *ZO-1*, *JAM-A*, *occludin, Claudin-1*, and *Claudin-4* in the colonic mucosa compared with low-level RPG supplementation. This discrepancy may be related to the supplementation level, as 200 g/day RPG represented approximately 2.5% of dietary dry matter in the previous study, compared with approximately 3% in the present study. Notably, high-glucose intake has been shown in mice to alter the gut microbiota, increase intestinal permeability, and reduce the expression of TJ proteins, including *ZO-1* and *occludin* [16,29]. Thus, the adverse effects observed with high-level RPG may be associated with excessive glucose intake and subsequent impairment of intestinal barrier function. These findings highlight the importance of an appropriate dietary glucose level for intestinal health and suggest that, under the present experimental conditions, 1% RPG was more favorable for maintaining colonic physical barrier integrity. Taurine is highly enriched in the small intestine and has been widely recognized for its protective effects on intestinal mucosal integrity in several animal species, including weaned piglets [27]. However, its effects may depend on the supplementation level. In weaned pigs, moderate taurine supplementation has been associated with beneficial effects on intestinal function, whereas high taurine supplementation (1.5% and 3%) increased IL-6, TNF-α, and Caspase-3 production, upregulated *Caspase-3* expression, and inhibited GLP-2 secretion, suggesting that excessive taurine may induce inflammatory and apoptotic responses and adversely affect intestinal function [17]. Consistent with this dose-dependent response, supplementation with 0.4% RPT produced greater growth-promoting effects than 0.6% RPT in heat-stressed sheep, while 0.4% RTP also suppressed pro-inflammatory gene expression in the jejunum through pathways such as JAK-STAT and improved the jejunal bacterial community [12]. In the present study, high-level RPT supplementation decreased the expression of *Claudin-1* and *JAM-A* in the colon, suggesting that excessive taurine supplementation may compromise colonic barrier function. Furthermore, significant interactions between RPG and RPT were observed for several TJ proteins, indicating that the effects of glucose and taurine on intestinal barrier function were not independent. Previous research has also demonstrated interactions between RPG and RPT in hepatic glucose and bile acid metabolism in yaks [6]. Although the mechanisms underlying the RPG–RPT interaction on colonic barrier function remain unclear, these findings suggest that the effects of taurine may be influenced by the dietary metabolic environment.

DAO is predominantly expressed in intestinal epithelial cells and is widely used as a sensitive indicator of intestinal mucosal integrity and epithelial injury [30,31]. Previous studies have shown that dietary taurine supplementation decreases serum DAO concentrations in weaned piglets, suggesting improved intestinal barrier function [32]. In contrast, high-level RPT supplementation in the present study significantly decreased both DAO activity and content in the colonic mucosa, which may indicate impaired mucosal integrity and increased intestinal permeability. This interpretation is further supported by the concomitant downregulation of multiple TJ proteins.

### 4.3. Effects of RPG and RPT on Colonic Chemical Barrier Function in Yaks

MUC2 and LZM are essential components of the intestinal chemical barrier, serving critical roles in protecting the intestinal mucosa against pathogenic microorganisms and other luminal insults while maintaining mucosal defense and immune homeostasis [33]. MUC2, the principal secretory product of goblet cells, constitutes the major structural component of the intestinal mucus layer.

As a key protective glycoprotein, MUC2 forms the first line of defense against harmful luminal stimuli and contributes substantially to the maintenance of mucosal integrity under physiological conditions [34]. Reduced MUC2 secretion has been reported in patients with inflammatory bowel disease, resulting in thinning of the mucus layer and increased susceptibility to mucosal injury [35]. In the present study, high-level RPT supplementation significantly decreased MUC2 content in the colonic mucosa, indicating impaired mucus barrier function. MUC2 has been shown to act synergistically with antimicrobial peptides to inhibit pathogen colonization, reinforce antimicrobial defense, and maintain intestinal microbial homeostasis [36]. Consistent with the reduction in MUC2, our previous results demonstrated that high-level RPT supplementation increased the relative abundance of Verrucomicrobiota, a bacterial phylum associated with mucin degradation. These findings suggest that excessive RPT supplementation may compromise the colonic mucus barrier by reducing MUC2 production, thereby promoting alterations in the intestinal microbiota and increasing susceptibility to mucosal injury. Collectively, these results indicate that an appropriate level of RPG and RPT supplementation is beneficial for maintaining intestinal barrier function and immune homeostasis, whereas excessive RPT may exert detrimental effects.

LZM is an important antimicrobial effector of the innate immune system [37]. In addition to its direct bacteriolytic activity, LZM participates in the regulation of inflammatory signaling pathways and other immune-related biological processes [38]. Previous studies have demonstrated that dietary taurine supplementation enhances innate immune responses in aquatic animals. For example, supplementation with 8 g/kg taurine significantly increased hepatic LZM, alkaline phosphatase, and acid phosphatase activities in Sebastes schlegelii, although these beneficial effects gradually diminished as the taurine level increased, suggesting that excessive taurine supplementation may attenuate immune function [39]. Similarly, Zhang et al. [40] reported that taurine supplementation alleviated acute ammonia toxicity in Pelteobagrus fulvidraco by preventing declines in LZM activity and phagocytic capacity. These observations collectively imply that taurine exerts dose-dependent immunomodulatory effects. In contrast, under the present experimental conditions, high-level RPT supplementation did not significantly affect colonic LZM content, indicating that the adverse effects of excessive RPT on the colonic chemical barrier were primarily associated with impaired mucus secretion rather than altered lysozyme production.

### 4.4. Effects of RPG and RPT on Colonic Immune Barrier Function in Yaks

The intestinal immune barrier is primarily composed of gut-associated lymphoid tissue, including mesenteric lymph nodes, isolated lymphoid follicles, Peyer’s patches, and immune effector cells distributed throughout the intestinal epithelium and lamina propria [41]. These lymphoid tissues produce a variety of immunoglobulins, among which sIgA is the predominant antibody responsible for maintaining mucosal immune defense. As the first line of adaptive immunity at the mucosal surface, sIgA prevents pathogen adhesion and invasion while contributing to intestinal immune homeostasis. Previous studies have demonstrated that dietary taurine supplementation at 0.05–0.20% of dry matter increases ileal mucosal sIgA concentrations in broilers [42], with similar responses reported in laying hens [43] and meat ducks [44]. In contrast, the present study showed that increasing dietary RPT supplementation significantly reduced colonic sIgA content. This reduction suggests that excessive RPT supplementation suppresses immunoglobulin secretion, thereby weakening mucosal defense against pathogenic microorganisms and compromising hindgut immune barrier function.

IL-10 is a pleiotropic anti-inflammatory cytokine that plays a central role in maintaining intestinal immune tolerance. Deficiency in either *IL-10* or its receptor results in severe spontaneous colitis, highlighting its indispensable role in intestinal immune homeostasis [45]. Previous studies have shown that supplementation with 0.1% taurine effectively attenuates lipopolysaccharide-induced intestinal inflammation in weaned piglets [46]. However, excessive taurine intake has been reported to increase the expression of pro-inflammatory cytokines, impair liver function, and reduce immune enzyme activity in fish [47]. In the present study, a significant interaction between RPG and RPT levels was observed for colonic *IL-10* mRNA expression, with the highest expression observed in the LGLT and HGHT groups. This interaction suggests that the effects of RPG on *IL-10* expression may depend on the supplementation level of RPT, and vice versa. These findings contrast with previous studies demonstrating the beneficial immunomodulatory effects of moderate RPG supplementation. For example, RPG supplementation reduced postpartum inflammatory responses in periparturient dairy cows [48], while supplementation with 25 g/kg RPG downregulated the pro-inflammatory cytokines *IL-17A*, *IL-17F*, and *IL-22* [9].

Interestingly, a notable finding was observed in the present study. Although *IL-10* is a key anti-inflammatory cytokine that functions as a negative-feedback regulator, its expression pattern paralleled that of the pro-inflammatory cytokines *TNF-α*, *IL-6*, and *IL-1β*, with the highest levels detected in the low-RPG and low-RPT groups and significantly lower levels in the high-RPG and high-RPT groups. This pattern may reflect the induction of *IL-10* secretion by moderate inflammatory stimulation as part of a physiological negative-feedback mechanism [49], thereby preserving intestinal mucosal integrity [50]. Consistent with this interpretation, the expression of tight junction proteins, MUC2, DAO, and sIgA was also highest in the LGLT group. In contrast, although the HGHT group exhibited markedly reduced levels of pro-inflammatory cytokines, the simultaneous decrease in IL-10, whose expression is also dependent on immune cell activation, may indicate a generalized suppression of mucosal immune activity rather than a beneficial resolution of inflammation [51,52]. Collectively, these findings suggest that the immunomodulatory effects of both RPG and RPT are dose-dependent. Appropriate supplementation appears to enhance intestinal anti-inflammatory capacity, whereas excessive supplementation may impair immune function and compromise intestinal immune homeostasis. Furthermore, a significant interaction between RPG and RPT was observed for *IL-10* mRNA expression, with the LGLT treatment exhibiting the highest level of colonic *IL-10* expression, suggesting that an appropriate combination of glucose and taurine provides the greatest benefit for maintaining hindgut immune function.

### 4.5. Effects of RPG and RPT on Colonic Microbial Barrier in Yaks

In the present study, 16S rRNA gene sequencing was used to characterize the colonic microbial of yaks receiving different levels of RPG and RPT. The rarefaction curves approached saturation and Good’s coverage exceeded 98%, indicating adequate sequencing depth. High-level RPG and RPT supplementation reduced bacterial richness and diversity, as indicated by lower observed ASV, Chao1, ACE, and Shannon indices, while beta diversity analysis showed distinct microbial community clustering among treatment groups. Firmicutes, Proteobacteria, and Bacteroidota were the dominant bacterial phyla, consistent with previous reports in ruminants and other mammals [53,54]. Notably, Proteobacteria are metabolically and ecologically responsive to changes in intestinal conditions and may increase under dysbiotic states [55,56,57]. These findings indicate that dietary RPG and RPT levels can alter the diversity and composition of the yak colonic microbiota.

At the phylum level, high-level RPG supplementation significantly decreased the relative abundance of Proteobacteria, consistent with our previous observations in the rumen [15]. Proteobacteria comprise a metabolically diverse group of microorganisms capable of degrading a wide range of dietary substrates in the gastrointestinal tract of ruminants [58]. Their abundance has been reported to be negatively associated with dietary fiber content in both dairy cows and humans [59,60], and enrichment of Proteobacteria has been proposed to facilitate fiber degradation in buffalo [61]. Moreover, members of this phylum possess broad metabolic versatility that may enhance nutrient utilization and energy harvest under challenging environmental conditions, such as cold exposure [62]. Therefore, the reduction in Proteobacteria abundance observed in the high-RPG group may have diminished the capacity for fiber utilization. High-level RPT supplementation, in contrast, significantly increased the relative abundance of Verrucomicrobiota in the colon. Members of this phylum are characterized by their ability to degrade mucin, a major structural component of the intestinal mucus layer that plays a critical role in protecting epithelial cells from microbial invasion [63]. Excessive mucin degradation may weaken the mucus barrier, increase epithelial exposure to luminal microorganisms, and ultimately compromise intestinal barrier function. This interpretation is supported by the concurrent reduction in colonic MUC2 content observed in the present study. Collectively, these findings suggest that excessive RPT supplementation may impair mucosal barrier integrity by promoting mucin-degrading bacteria, whereas an appropriate level of RPT is more favorable for maintaining intestinal barrier function.

At the genus level, *Ruminococcaceae_UCG-005*, *Ruminococcaceae_UCG-010*, *Rikenellaceae_RC9_gut_group*, and *[Eubacterium] coprostanoligenes group* were identified as the predominant bacterial taxa in the yak colon, consistent with the findings of Chang et al. [64]. *Ruminococcaceae* members are known to degrade plant polysaccharides and cellulose, thereby contributing to energy harvest through microbial fermentation [65]. An increase in the abundance of *Ruminococcaceae* has also been reported in black howler monkeys during periods of energy deprivation, suggesting that these bacteria may compensate for reduced energy intake by enhancing fiber fermentation efficiency [66]. In the present study, high-level RPG diet increased the relative abundance of *Ruminococcaceae*, which may indicate enhanced utilization of dietary fiber. A similar trend was previously observed following RPG supplementation in ruminants [37]. *Christensenellaceae_R-7_group*, a representative member of the family *Christensenellaceae*, is widely distributed in the intestinal tract of humans and animals and has been consistently associated with intestinal health. Reduced abundance of this taxon has been reported in individuals with gastrointestinal disorders [67], whereas studies in ruminants have suggested that it promotes gastrointestinal development and enhances nutrient digestion and absorption [68,69]. In the present study, high-level RPG supplementation decreased the relative abundance of *Christensenellaceae_R-7_group*, whereas high-level RPT supplementation produced the opposite response.

Significant interactions between RPG and RPT levels were observed for the relative abundances of *Rikenellaceae_RC9_gut_group* and *Bacteroides*, with both taxa showing the highest abundances under the high-glucose treatment. *Bacteroides* are Gram-negative, obligate anaerobic commensals that contribute to polysaccharide degradation, nutrient utilization, and colonization resistance against enteric pathogens [70,71]. However, some Bacteroides species can act as opportunistic pathogens when the intestinal barrier is compromised [72]. Therefore, the increased abundance of *Bacteroides* may reflect alterations in the intestinal microbial ecosystem rather than a uniformly beneficial response. This interpretation is consistent with the reduced expression of tight junction proteins and IL-10 observed in the same treatment group. The *Rikenellaceae_RC9_gut_group* belongs to the family Rikenellaceae and has been associated with lipid metabolism. Previous studies have reported increased abundance of this taxon in mice fed a high-fat diet supplemented with high-dose genistein [73]. Thus, the enrichment of the *Rikenellaceae_RC9_gut_group* may reflect alterations in colonic lipid metabolism. Overall, RPG and RPT altered the composition of the colonic microbiota in a level-dependent manner. High-level supplementation was associated with changes in bacterial taxa related to carbohydrate and lipid metabolism and coincided with impaired intestinal barrier and immune function. In contrast, the LGLT treatment was associated with a relatively balanced microbial profile and preserved intestinal barrier function, suggesting that the combination of lower RPG and RPT levels may be more favorable for maintaining hindgut health in yaks.

## 5. Conclusions

This study demonstrated that dietary supplementation with rumen-protected glucose and rumen-protected taurine exerted dose-dependent effects on hindgut health in yaks. Excessive supplementation of rumen-protected glucose impaired colonic physical barrier integrity by reducing the expression of tight junction proteins and altering microbial diversity, whereas excessive rumen-protected taurine weakened chemical and immune barrier functions by decreasing diamine oxidase activity, mucin production, secretory immunoglobulin A concentration, and anti-inflammatory responses. In contrast, moderate supplementation maintained better hindgut barrier function and microbial homeostasis. Among the dietary treatments, the combination of low-level rumen-protected glucose and low-level rumen-protected taurine exhibited the most favorable effects on maintaining physical, chemical, and immune barrier functions, as well as a balanced microbial community. These findings indicate that the biological effects of rumen-protected glucose and rumen-protected taurine are highly dependent on supplementation levels and provide valuable insights for optimizing nutritional strategies to promote intestinal health in yaks.

## Figures and Tables

**Table 1:** Composition and nutrient levels of experimental diet (% DM basis).

Item	Treatment ^1^			
Ingredients, % DM	LGLT	LGHT	HGLT	HGHT
Corn	15.5	15.5	14.7	14.7
Wheat bran	5.6	5.6	5.0	5.0
Soybean meal	9.0	9.0	9.4	9.4
Rapeseed meal	3.0	3.0	3.0	3.0
Palm oil	2.0	2.0	1.0	1.0
CaHPO_4_	0.1	0.1	0.1	0.1
CaCO_3_	0.6	0.6	0.6	0.6
NaCl	0.2	0.2	0.2	0.2
Premix ^2^	1.0	1.0	1.0	1.0
Rumen protection glucose	1.0	1.0	3.0	3.0
Leymus chinensis hay	27.0	27.0	27.0	27.0
Straw	35.0	35.0	35.0	35.0
Nutrient levels ^3^, %				
NEm MJ/kg	5.97	5.97	5.97	5.97
NEg MJ/kg	3.48	3.48	3.48	3.48
CP	9.88	9.88	9.88	9.88
NDF	41.24	41.24	41.31	41.31
ADF	19.90	19.90	20.33	20.33
Ca	0.42	0.42	0.42	0.42
P	0.24	0.24	0.23	0.23

NEm = net energy for maintained; NEg = net energy for gain; NDF = neutral detergent fiber; ADF = acid washing fiber; CP = crude protein; Ca = calcium; P = phosphorus. ^1^ LGLT = 1.0% RPG + 5 g/day RPT; LGHT = 1.0% RPG + 20 g/day RPT; HGLT = 3.0% RPG + 5 g/day RPT; HGHT LT = 3.0% RPG + 20 g/day RPT. ^2^ The premix provides Cu—10 mg, Fe—40 mg, Mn—60 mg, Zn—60 mg, Se—0.3 mg, I—0.5 mg, Co—0.1 mg, VA—4400 IU, VD_3_—1300 IU, and V— 20 IU. ^3^ NEg and NEm were calculated from data provided by the Nutrient Requirements of Beef Cattle [19], and other values were analyzed.

**Table 2:** Primer sequences for real-time PCR.

Gene	Primer Sequence (5′–3′)	Length (bp)	Accession Number
*ZO-1*	F: CCGAATGAAACCGCACACAAACC R: GTCTCCACGCCACTGTCAAACTC	107	XM_014476599.1
*Occludin*	F: GCCTGTGTTGCCTCCACTCTTG R: CCATAGCCATAACCGTAGCCATAGC	143	XM_005889348.2
*Claudin-1*	F: CCCGTGCCTTGATGGTGATTGG R: CATCTTCTGTGCCTCGTCGTCTTC	110	XM_005897671.2
*Claudin-4*	F: TCATCGGCAGCAACATCGTCAC R: CAGCAGCGAGTCGTACACCTTG	110	XM_005892850.2
*JAM-A*	F: GTGCCTCCATCCAAGCCTACAATC R: GGCATCTCTACTCCATCCTTGAACC	134	XM_010802736.3
*IL-10*	F: GAACCACGGGCCTGACATCAAG R: CTTCTCCACCGCCTTGCTCTTG	127	XM_005891650
*IL-1β*	F: CTCCGACGAGTTTCTGTGTGACG R: GAGAGGAGGTGGAGAGCCTTCAG	120	NM_174093.1
*IL-6*	F: CACTGACCTGCTGGAGAAGATGC R: CCGAATAGCTCTCAGGCTGAACTG	115	XM_005901249.2
*TNF-α*	F: TGAAGGAAGAGGAGAGGCTCATCG R: GTGGTCATCGGAGTTGCTGGTG	107	XM_005904178.1
*NF-κB*	F: GCCTGCTGAATGCTCTGTCTGAC R: CTCTGTTTCCTGTTCCACCGACTG	143	XM_005887214.2
*p38 MAPK*	F: TGCTGGAGAAGATGCTTGTATTGG R: TCGTCGTCAGGATCGTGGTAC	94	XM_005890506.2
*JNK*	F: CAGAAGCAAGCGTGACAGCA R: TTCCTTGGGCTCCTGAACCT	130	XM_005900620.1
*ERK*	F: AGGTGTGGTGTTCAAGGTCTCC R: TGATCTCGCCGTCGCTGTAG	100	XM_005893283.2
*β-Actin*	F: TGGCGCTTGACTCAGGATTT R: CAATCAAGTCCTCGGCCACA	102	XM_005887322.2

*ZO-1* = zonula occludens-1; *JAM-A* = junctional adhesion molecule A; *NF-κB* = nuclear factor kappa B; *JNK* = c-Jun N-terminal kinase; *ERK* = extracellular signal-regulated kinase; *p38 MAPK* = p38 mitogen-activated protein kinase; *TNF-α* = tumor necrosis factor-alpha; *IL-6* = interleukin-6; *IL-1β* = interleukin-1 beta; *IL-10* = interleukin-10.

**Table 3:** Effects of RPG and RPT on growth performance in yaks.

Item	LG		HG		SEM	*p*-Values ^1^		
	LT	HT	LT	HT		G	T	G × T
DMI	4.28	4.26	4.36	4.28	0.05	0.660	0.630	0.757
ADG	0.40	0.36	0.38	0.36	0.02	0.772	0.423	0.796
F/G	11.91	12.13	11.86	12.32	0.51	0.950	0.754	0.913

LG = 1% RPG; HG = 3% RPG; LT = 5 g/d RPT; HT = 20 g/d RPT; ADG = average daily gain; DMI = dry matter intake; F/G = feed-to-gain ratio. ^1^ *P*_G_, main effect of dietary RPG level; *P*_T_, main effect of RPT supplementation level; *P*_G×T_, interaction between dietary RPG level and RPT supplementation level.

**Table 4:** Effects of RPG and RPT on the mRNA expression of genes related to physical and immune barrier functions in yaks.

Item	Gene	LG		HG		SEM	*p*-Values ^1^		
		LT	HT	LT	HT		G	T	G × T
Physical barrier function	*ZO-1*	1.00	1.01	0.84	0.72	0.06	0.087	0.575	0.505
	*Occludin*	1.00 a	0.72 b	0.41 b	0.49 b	0.02	<0.001	0.121	0.010
	*Claudin-1*	1.00	0.53	0.57	0.24	0.07	<0.001	<0.001	0.601
	*Claudin-4*	1.00	0.66	0.49	0.51	0.06	0.003	0.129	0.070
	*JAM-A*	1.00	0.71	0.62	0.59	0.05	0.006	0.051	0.146
Immune barrier function	*NF-* *κ* *B*	1.00	0.88	0.84	0.61	0.04	0.002	<0.001	0.747
	*JNK*	1.00	0.61	0.66	0.39	0.05	0.285	0.176	0.483
	*ERK*	1.00	0.92	0.96	0.71	0.06	0.017	0.016	0.337
	*p38 MAPK*	1.00	1.00	0.82	0.92	0.06	0.201	0.533	0.533
	*TNF-* *α*	1.00	0.66	0.41	0.30	0.07	<0.001	<0.001	0.278
	*IL-6*	1.00	0.47	0.49	0.16	0.07	<0.001	<0.001	0.291
	*IL-1* *β*	1.00	0.45	0.39	0.15	0.08	<0.001	<0.001	0.138
	*IL-10*	1.00 a	0.22 b	0.22 b	0.14 b	0.08	<0.001	<0.001	<0.001

Treatment: LGLT = 1.0% RPG + 5 g/day RPT; LGHT = 1.0% RPG + 20 g/day RPT; HGLT = 3.0% RPG + 5 g/day RPT; HGHT LT = 3.0% RPG + 20 g/day RPT. LG = 1% RPG; HG = 3% RPG; LT = 5 g/d RPT; HT = 20 g/d RPT; *ZO-1* = zonula occludens-1; *JAM-A* = junctional adhesion molecule A; *NF-κB* = nuclear factor kappa B; *JNK* = c-Jun N-terminal kinase; *ERK* = extracellular signal-regulated kinase; *p38 MAPK* = p38 mitogen-activated protein kinase; *TNF-α* = tumor necrosis factor-alpha; *IL-6* = interleukin-6; *IL-1β* = interleukin-1 beta; *IL-10* = interleukin-10. ^1^ *P*_G_, main effect of dietary RPG level; *P*_T_, main effect of RPT supplementation level; *P*_G×T_, interaction between dietary RPG level and RPT supplementation level. Different lowercase letters within a row indicate significant differences (*p* < 0.05).

**Table 5:** Effects of RPG and RPT on α-diversity of the colonic microbiota in yaks.

Item	LG		HG		SEM	*p*-Values ^1^		
	LT	HT	LT	HT		G	T	G × T
Ace	1264.23	1244.98	1223.25	989.49	38.58	0.040	0.076	0.128
Chao	1269.33	1244.52	1221.72	991.26	38.66	0.037	0.074	0.145
Shannon	6.08	5.97	5.85	5.88	0.03	0.007	0.446	0.221
Simpson	0.99	0.99	0.99	0.99	0.00	0.240	0.793	0.060
PD	88.47	91.84	88.53	75.29	2.28	0.055	0.240	0.054

Treatment: LGLT = 1.0% RPG + 5 g/day RPT; LGHT = 1.0% RPG + 20 g/day RPT; HGLT = 3.0% RPG + 5 g/day RPT; HGHT LT = 3.0% RPG + 20 g/day RPT. LG = 1% RPG; HG = 3% RPG; LT = 5 g/d RPT; HT = 20 g/d RPT. ^1^ G, main effect of dietary RPG level; T, main effect of RPT supplementation level; G × T, interaction between dietary RPG level and RPT supplementation level.

**Table 6:** Effects of RPG and RPT on the relative abundance of colonic bacterial phyla and genera in yaks.

Item	Bacteria	LG		HG		SEM	*p*-Values ^1^		
		LT	HT	LT	HT		G	T	G × T
Phylum level	Firmicutes	69.09	66.15	66.30	67.30	0.98	0.686	0.635	0.339
	Bacteroidetes	24.33	24.58	27.80	24.96	1.03	0.372	0.547	0.473
	Proteobacteria	2.43	3.30	2.26	1.96	0.18	0.024	0.369	0.075
	Verrucomicrobia	0.58	1.11	0.59	1.63	0.16	0.371	0.012	0.380
	Chloroflexi	0.60	0.87	0.67	0.45	0.08	0.235	0.856	0.113
Genus level	*Ruminococcaceae UCG-005*	14.36	12.56	15.65	16.12	0.52	0.017	0.487	0.241
	*[Eubacterium] coprostanoligenes group*	7.06	6.89	8.06	7.44	0.31	0.232	0.542	0.723
	*Ruminococcaceae UCG-010*	5.70 b	5.46 b	6.10 b	7.30 a	0.20	0.001	0.138	0.031
	*Ruminococcaceae UCG-013*	6.65	5.95	5.46	6.36	0.21	0.338	0.810	0.061
	*Rikenellaceae RC9 gut group*	5.27 bc	6.11 ab	4.49 c	7.29 a	0.30	0.668	<0.001	0.047
	*Bacteroides*	3.21 b	4.06 b	7.39 a	3.92 b	0.52	0.033	0.157	0.024
	*Christensenellaceae R-7 group*	4.36	5.29	3.09	4.41	0.21	0.002	0.002	0.542
	*Prevotellaceae UCG-003*	1.63	2.20	3.32	1.88	0.35	0.334	0.538	0.158
	*Ruminococcaceae UCG-009*	1.76	2.02	1.98	2.02	0.08	0.506	0.362	0.501
	*Prevotellaceae UCG-004*	1.66	0.91	2.14	2.00	0.23	0.090	0.328	0.492

Treatment: LGLT = 1.0% RPG + 5 g/day RPT; LGHT = 1.0% RPG + 20 g/day RPT; HGLT = 3.0% RPG + 5 g/day RPT; HGHT LT = 3.0% RPG + 20 g/day RPT. LG = 1% RPG; HG = 3% RPG; LT = 5 g/d RPT; HT = 20 g/d RPT. ^1^ G, main effect of dietary RPG level; T, main effect of RPT supplementation level; G × T, interaction between dietary RPG level and RPT supplementation level. Different lowercase letters within a row indicate significant differences (*p* < 0.05).

## Data Availability

The original contributions presented in this study are included in this article. Further inquiries can be directed to the corresponding author.

## Abbreviations

The following abbreviations are used in this manuscript:

ADG	Average daily gain
ASV	Amplicon sequence variant
DAO	Diamine oxidase
DM	Dry matter
DMI	Dry matter intake
*ERK*	Extracellular signal-regulated kinase
*IL-1β*	Interleukin-1 beta
*IL-6*	Interleukin-6
*IL-10*	Interleukin-10
*JAM-A*	Junctional adhesion molecule A
*JNK*	c-Jun N-terminal kinase
F/G	Feed-to-gain ratio
LZM	Lysozyme
MUC2	Mucin-2
*NF-κB*	Nuclear factor kappa B
*p38 MAPK*	p38 mitogen-activated protein kinase
RPG	Rumen-protected glucose
RPT	Rumen-protected taurine
sIgA	Secretory immunoglobulin A
*TNF-α*	Tumor necrosis factor-alpha
*ZO-1*	Zonula occludens-1

## References

[1] Yao X., Kausar T., Wang W., Xu C., Liu H., Ahmad A.A., Wanapat M., Tahir R., Bai Y., Degen A.A. (2026). Feedlot diets improve yak meat quality by altering rumen microbiota and metabolic pathways. J. Agric. Food Res..

[2] Jing X., Ding L., Zhou J., Huang X., Degen A., Long R. (2022). The adaptive strategies of yaks to live in the Asian highlands. Anim. Nutr..

[3] Gao Z., Zhang F., Almasoudi S.H., Al-Zahrani M., Basri A.M., Makhlof R.T., Yu Z., Gui L. (2026). Expression profiles and their influence on intramuscular fat in yaks at two developmental stages role of RNA sequencing. Protoplasma.

[4] Zhang C., Wang J., Zhao L., Xu S., Wang X., Zhao X. (2026). Transformative changes of pastoral livestock production and its consequences on the Qinghai–Xizang Plateau. Grassl. Res..

[5] Zhao S., Zhou J., Guan S., Wang X., Wen X., Zhao K., Yang H., Lu L., Zhang B., Chen Y. (2025). Dietary energy and protein gradients drive metabolic adaptation in growing-finishing yaks on the Qinghai-Tibet Plateau. Anim. Nutr..

[6] Chen Y., Wang X., Lu L., Zhang B., Yang H., Zhao S., Wang Z., Wang L., Peng Q., Xue B. (2025). Effects of Dietary Rumen-Protected Glucose and Rumen-Protected Taurine Levels on Growth Performance, Serum Biochemical Indicators, and Liver Health in Yaks. Animals.

[7] Bao Y., Zhou J., Yang X., Shi R., Liao Y. (2025). Effects of Forage-to-Concentrate Ratio During Cold-Season Supplementation on Growth Performance, Serum Biochemistry, Hormones, and Antioxidant Capacity in Yak Calves on the Qinghai–Tibet Plateau. Animals.

[8] Zhang F., Wang Y., Tang X., Xiong B. (2025). Changes in milk fatty acids and metabolites in dairy cows supplemented with varying levels of rumen-protected glucose during early lactation. J. Dairy Sci..

[9] Zhang X., Li X., Wu J., Jiao J., He Z., Tan Z., Han X. (2021). Rumen-protected glucose supplementation in transition dairy cows shifts fermentation patterns and enhances mucosal immunity. Anim. Nutr..

[10] Chen J., Ma L., Zhang Z., An F., Li X., Li B., An T., Wang L. (2026). Rumen-Protected Glucose Supplementation Enhances Yak Calf Growth Through Gut Microbiota–Metabolic Interactions. Animals.

[11] Zhou M., Wu Z., Deng D., Wang B., Zhou X., Zhou B., Wang C., Zeng Y. (2024). Effects of taurine on the growth performance, diarrhea, oxidative stress and intestinal barrier function of weanling piglets. Front. Vet. Sci..

[12] Wei Y., Zhang Y., Liu X., Chen X., Lu G., Wang Y., Hu X., Ouyang K. (2026). Rumen-Protected Taurine Alleviates Heat Stress Injury in Hu Sheep by Regulating Inflammatory Response, Gut Microbiota and Transcriptome. Animals.

[13] Yu H., Guo Z., Shen S., Shan W. (2016). Effects of taurine on gut microbiota and metabolism in mice. Amino Acids.

[14] Wang X., Zhao K., Zhao S., Zhou J., Cao M., Lu L., Chen Y., Yang H., Zhang B., Shao C. (2024). Effects of dietary rumen-protected glucose level and taurine supplementation on weight change and oxidative stress state of yaks after transport. Front. Vet. Sci..

[15] Cao M., Zhao K., Zhao S., Wang X., Zhou J., Lu L., Chen Y., Yang H., Zhang B., Shao C. (2026). Effects of dietary rumen-protected glucose and taurine levels on growth performance, rumen fermentation, apparent nutrient digestibility, and rumen microbiota in yaks. Anim. Feed Sci. Technol..

[16] Do M.H., Lee E., Oh M., Kim Y., Park H. (2018). High-glucose or-fructose diet cause changes of the gut microbiota and metabolic disorders in mice without body weight change. Nutrients.

[17] Liu Y., Mao X., Yu B., He J., Zheng P., Yu J., Luo J., Chen D. (2014). Excessive dietary taurine supplementation reduces growth performance, liver and intestinal health of weaned pigs. Livest. Sci..

[18] (2004). Feeding Standard of Beef Cattle.

[19] NASEM (2016). Nutrient Requirements of Beef Cattle.

[20] AOAC (2005). Official Methods of Analysis.

[21] AOAC (1990). Official Methods of Analysis.

[22] Van Soest P.V., Robertson J.B., Lewis B.A. (1991). Methods for dietary fiber, neutral detergent fiber, and nonstarch polysaccharides in relation to animal nutrition. J. Dairy Sci..

[23] Van Keulen J., Young B.A. (1977). Evaluation of acid-insoluble ash as a natural marker in ruminant digestibility studies. J. Anim. Sci..

[24] Hansson G.C., Johansson M.E. (2010). The inner of the two Muc2 mucin-dependent mucus layers in colon is devoid of bacteria. Gut Microbes.

[25] Zihni C., Mills C., Matter K., Balda M.S. (2016). Tight junctions: From simple barriers to multifunctional molecular gates. Nat. Rev. Mol. Cell Biol..

[26] Wouda S.H., Geertsema S., van Goor H., Dijkstra G., Faber K.N., Bourgonje A.R. (2026). Advances in blood-based biomarkers of gut barrier integrity. Trends Mol. Med..

[27] Wen C., Guo Q., Wang W., Duan Y., Li F. (2020). Taurine Alleviates Intestinal Injury by Mediating Tight Junction Barriers in Diquat-Challenged Piglet Models. Front. Physiol..

[28] Zhang Z.J.J. (2019). Effects of rumen-protected glucose on ileal microbiota and genes involved in ileal epithelial metabolism and immune homeostasis in transition dairy cows. Anim. Feed Sci. Technol..

[29] Zhang X., Monnoye M., Mariadassou M., Beguet-Crespel F., Lapaque N., Heberden C., Douard V. (2021). Glucose but not fructose alters the intestinal paracellular permeability in association with gut inflammation and dysbiosis in mice. Front. Immunol..

[30] Yang S., Fan L., Yin L., Zhao Y., Li W., Zhao R., Jia X., Dong F., Zheng Z., Zhao D. (2025). Ginseng exosomes modulate M1/M2 polarisation by activating autophagy and target IKK/IĸB/NF-ĸB to alleviate inflammatory bowel disease. J. Nanobiotechnol..

[31] Jia R., Han Y., Zhu Q., Zhang J., Zhang H., Ka M., Ma Y., Gamah M., Zhang W. (2025). Activation of notch signaling pathway is a potential mechanism for mucin2 reduction and intestinal mucosal barrier dysfunction in high-altitude hypoxia. Sci. Rep..

[32] Chen J., Zhang X., Zhang Y., Jiang S., Han Y., Zhang L., Zhang Y., Du H. (2024). Taurine enhances growth performance by improving intestinal integrity and antioxidant capacity of weaned piglets. J. Anim. Sci..

[33] Xu X., Li W., Yu Z., Zhang L., Duo T., Zhao Y., Qin W., Yang W., Ma L. (2022). Berberine Ameliorates Dextran Sulfate Sodium-Induced Ulcerative Colitis and Inhibits the Secretion of Gut Lysozyme via Promoting Autophagy. Metabolites.

[34] Fan F., Guo R., Pan K., Xu H., Chu X. (2025). Mucus and mucin: Changes in the mucus barrier in disease states. Tissue Barriers.

[35] Antoni L., Nuding S., Wehkamp J., Stange E.F., Stuttgart F.B.I.O., Tübingen U.O., Department O.I.M.I., Robert-Bosch-Hospital (2014). Intestinal barrier in inflammatory bowel disease. World J. Gastroenterol..

[36] Cobo E.R., Chadee K., Kissoon-Singh V., Moreau F. (2015). Colonic MUC2 mucin regulates the expression and antimicrobial activity of beta-defensin 2. Mucosal Immunol..

[37] Mukherjee S., Vaishnava S., Hooper L.V. (2008). Multi-layered regulation of intestinal antimicrobial defense. Cell. Mol. Life Sci..

[38] Gao X., Cao Q., Cheng Y., Zhao D., Wang Z., Yang H., Wu Q., You L., Wang Y., Lin Y. (2018). Chronic stress promotes colitis by disturbing the gut microbiota and triggering immune system response. Proc. Natl. Acad. Sci. USA.

[39] Wei L. (2022). Effects of Dietary DL-Carnitine and Taurine Supplementation Ongrowth Performance, Antioxidant Ability and Stress Resistance of *Sebastes schlegel*. Master’s Thesis.

[40] Zhang M., Li M., Wang R., Qian Y. (2018). Effects of acute ammonia toxicity on oxidative stress, immune response and apoptosis of juvenile yellow catfish Pelteobagrus fulvidraco and the mitigation of exogenous taurine. Fish Shellfish Immunol..

[41] Brandtzaeg P., Kiyono H., Pabst R., Russell M.W. (2008). Terminology: Nomenclature of mucosa-associated lymphoid tissue. Mucosal Immunol..

[42] Oke O.E., Oni A.I., Akosile O.A., Oliyide K.M., Ishola C.A., Logunleko M.O., Jimoh O.A., Njoku C.P., Fasasi L.O., Uyanga V.A. (2025). Heat Stress and Gut Microbiome Dynamics in Poultry: Interplay, Consequences, and Mitigation Strategies. Anim. Res. One Health.

[43] Liu Q., Li W., Huang S., Zhao L., Zhang J., Ji C., Ma Q. (2022). R-is superior to S-form of α-lipoic acid in anti-inflammatory and antioxidant effects in laying hens. Antioxidants.

[44] Liu S., Zhang Q., Wang Y., Zhu W., Li L., Zhang Y., Zhang J. (2026). Dietary Scutellaria baicalensis polysaccharide as an antibiotic alternative improves growth performance, antioxidant capacity, and intestinal mucosal immunity in broiler chickens. Vet. World.

[45] Rogler G., Andus T. (1998). Cytokines in Inflammatory Bowel Disease. World J. Surg..

[46] Zhiru T., Jinyan L., Zhihong S., Jinlong L., Weizhong S., Junxia M., Yao W. (2018). Protective effects of taurine on growth performance and intestinal epithelial barrier function in weaned piglets challenged without or with lipopolysaccharide. Anim. Prod. Sci..

[47] Yan L.C., Feng L., Jiang W.D., Wu P., Liu Y., Jiang J., Tang L., Tang W., Zhang Y.A., Yang J. (2019). Dietary taurine supplementation to a plant protein source-based diet improved the growth and intestinal immune function of young grass carp (*Ctenopharyngodon idella*). Aquac. Nutr..

[48] Mccarthy C.S., Dooley B.C., Branstad E.H., Kramer A.J., Horst E.A., Mayorga E.J., Al-Qaisi M., Abeyta M.A., Perez-Hernandez G., Goetz B.M. (2020). Energetic metabolism, milk production, and inflammatory response of transition dairy cows fed rumen-protected glucose. J. Dairy Sci..

[49] Paul G., Khare V., Gasche C. (2012). Inflamed gut mucosa: Downstream of interleukin-10. Eur. J. Clin. Invest..

[50] Meyer F., Wendling D., Demougeot C., Prati C., Verhoeven F. (2023). Cytokines and intestinal epithelial permeability: A systematic review. Autoimmun. Rev..

[51] Glocker E., Kotlarz D., Boztug K., Gertz E.M., Schäffer A.A., Noyan F., Perro M., Diestelhorst J., Allroth A., Murugan D. (2009). Inflammatory bowel disease and mutations affecting the interleukin-10 receptor. N. Engl. J. Med..

[52] Saraiva M., O’Garra A. (2010). The regulation of IL-10 production by immune cells. Nat. Rev. Immunol..

[53] Hao W., Lee Y. (2004). Microflora of the gastrointestinal tract: A review. Public Health Microbiol. Methods Protoc..

[54] Zhang L., Jiang X., Li A., Waqas M., Gao X., Li K., Xie G., Zhang J., Mehmood K., Zhao S. (2020). Characterization of the microbial community structure in intestinal segments of yak (Bos grunniens). Anaerobe.

[55] Faith J.J., Guruge J.L., Charbonneau M., Subramanian S., Seedorf H., Goodman A.L., Clemente J.C., Knight R., Heath A.C., Leibel R.L. (2013). The long-term stability of the human gut microbiota. Science.

[56] Caporaso J.G., Lauber C.L., Costello E.K., Berg-Lyons D., Gonzalez A., Stombaugh J., Knights D., Gajer P., Ravel J., Fierer N. (2011). Moving pictures of the human microbiome. Genome Biol..

[57] Clemente-Postigo M., Queipo-Ortuño M.I., Murri M., Boto-Ordoñez M., Pérez-Martínez P., Andres-Lacueva C., Cardona F., Tinahones F.J. (2012). Endotoxin increase after fat overload is related to postprandial hypertriglyceridemia in morbidly obese patients. J. Lipid Res..

[58] Evans N.J., Brown J.M., Murray R.D., Getty B., Birtles R.J., Hart C.A., Carter S.D. (2011). Characterization of novel bovine gastrointestinal tract Treponema isolates and comparison with bovine digital dermatitis treponemes. Appl. Environ. Microbiol..

[59] Thoetkiattikul H., Mhuantong W., Laothanachareon T., Tangphatsornruang S., Pattarajinda V., Eurwilaichitr L., Champreda V. (2013). Comparative analysis of microbial profiles in cow rumen fed with different dietary fiber by tagged 16S rRNA gene pyrosequencing. Curr. Microbiol..

[60] De Filippo C., Cavalieri D., Di Paola M., Ramazzotti M., Poullet J.B., Massart S., Collini S., Pieraccini G., Lionetti P. (2010). Impact of diet in shaping gut microbiota revealed by a comparative study in children from Europe and rural Africa. Proc. Natl. Acad. Sci. USA.

[61] Pu X.X., Zhang X.M., Li Q.S., Wang R., Zhang M., Zhang S.Z., Lin B., Tan B., Tan Z.L., Wang M. (2023). Comparison of in situ ruminal straw fiber degradation and bacterial community between buffalo and Holstein fed with high-roughage diet. Front. Microbiol..

[62] Sun B., Wang X., Bernstein S., Huffman M.A., Xia D., Gu Z., Chen R., Sheeran L.K., Wagner R.S., Li J. (2016). Marked variation between winter and spring gut microbiota in free-ranging Tibetan Macaques (*Macaca thibetana*). Sci. Rep..

[63] Johansson M.E., Hansson G.C. (2016). Immunological aspects of intestinal mucus and mucins. Nat. Rev. Immunol..

[64] Liu C., Wu H., Liu S., Chai S., Meng Q., Zhou Z. (2019). Dynamic alterations in yak rumen bacteria community and metabolome characteristics in response to feed type. Front. Microbiol..

[65] Bergman E.N. (1990). Energy contributions of volatile fatty acids from the gastrointestinal tract in various species. Physiol. Rev..

[66] Amato K., Leigh S., Kent A., Mackie R., Yeoman C., Stumpf R., Wilson B., Nelson K., White B., Garber P. (2015). The gut microbiota appears to compensate for seasonal diet variation in the wild black howler monkey (*Alouatta pigra*). Microb. Ecol..

[67] Smirnova Y.D., Gryaznova M.V., Burakova I.Y., Syromyatnikov M.Y., Sviridova T.N., Lebedeva O.P., Maslov A.Y., Popov V.N. (2023). Study of microbiome aberrations in patients with irritable bowel syndrome with diarrhea by next-generation sequencing. Res. Results Biomed..

[68] Huang C., Ge F., Yao X., Guo X., Bao P., Ma X., Wu X., Chu M., Yan P., Liang C. (2021). Microbiome and metabolomics reveal the effects of different feeding systems on the growth and ruminal development of yaks. Front. Microbiol..

[69] Couch C.E., Arnold H.K., Crowhurst R.S., Jolles A.E., Sharpton T.J., Witczak M.F., Epps C.W., Beechler B.R. (2020). Bighorn sheep gut microbiomes associate with genetic and spatial structure across a metapopulation. Sci. Rep..

[70] Zafar H., Saier M.H. (2021). Gut Bacteroides species in health and disease. Gut Microbes.

[71] Cuskin F., Lowe E.C., Temple M.J., Zhu Y., Cameron E.A., Pudlo N.A., Porter N.T., Urs K., Thompson A.J., Cartmell A. (2015). Human gut Bacteroidetes can utilize yeast mannan through a selfish mechanism. Nature.

[72] Brook I. (2004). Intra-abdominal, retroperitoneal, and visceral abscesses in children. Eur. J. Pediatr. Surg..

[73] Zhou L., Xiao X., Zhang Q., Zheng J., Li M., Yu M., Wang X., Deng M., Zhai X., Li R. (2018). Improved glucose and lipid metabolism in the early life of female offspring by maternal dietary genistein is associated with alterations in the gut microbiota. Front. Endocrinol..

